# Shikonin promotes ubiquitination and degradation of cIAP1/2-mediated apoptosis and necrosis in triple negative breast cancer cells

**DOI:** 10.1186/s13020-021-00426-1

**Published:** 2021-02-01

**Authors:** Anqi Wang, Jiayu Liu, Yuhan Yang, Zhejie Chen, Caifang Gao, Zhanguo Wang, Chaomei Fu, Liang Zou, Shengpeng Wang

**Affiliations:** 1 School of medicine, Chengdu University, Chengdu, 610106 China; 2 State Key Laboratory of Quality Research in Chinese Medicine, Institute of Chinese Medical Sciences, University of Macau, Macao, China; 3 Clinical drug development, School of Medicine and Dentistry, Queen Mary University of London, London, UK; 4 School of Pharmacy, State Key Laboratory of Characteristic Chinese Medicine Resources in Southwest China, Chengdu University of Traditional Chinese Medicine, 611137 Chengdu, China; 5 Key Laboratory of Coarse Cereal Processing, Ministry of Agriculture and Rural Affairs, Chengdu University, 610106 Chengdu, China

**Keywords:** Shikonin, Breast cancer, Necrosis, Apoptosis, Ubiquitination

## Abstract

**Background:**

Shikonin (SKO) is a natural naphthoquinone derived from Chinese herbal medicine Arnebiae Radix with high development potentials due to its anti-inflammatory and anti-tumor activities. Overwhelming evidences have indicated that SKO can induce both necrosis and apoptosis in cancer cells, while the mechanisms for triple negative breast cancer cells is still need to be disclosed.

**Methods:**

In this study, kinds of molecular biological technologies, including flow-cytometry, Western blot, immunoprecipitation, enzyme-linked immunosorbent assay (ELISA) as well as real-time quantitative PCR (RT-qPCR), were applied for investigation on the underlying mechanisms of SKO induced necrosis and apoptosis for MDA-MB-231 cells. Inhibitors were also used for validation ofthe key signaling pathways involved in SKO triggered necrosis and apoptosis.

**Results:**

We found that SKO significantly triggered necrosis and apoptosis of MDA-MB-231 cells in both a concentration- and time-dependent manner. Mechanism studies demonstrated that SKO significantly promoted the autoubiquitination levels and facilitated the proteasome dependent degradation of cellular inhibitor of apoptosis protein 1 (cIAP1) and cIAP2 in MDA-MB-231 cells. Autoubiquitination and degradation of cIAP1 and cIAP2 induced by SKO further led to significant decreased ubiquitination and inactivation of RIP1, which played an important role in inhibition of pro-survival and accelerating of necrosis of MDA-MB-231 cells. Treatment with proteasome inhibitor lactacystin significantly rescued the cell viability induced by treatment of SKO.

**Conclusions:**

Our results demonstrate that SKO promotes the autoubiquitination and degradation of cIAP1 and cIAP2, which further induces the decrease of the ubiquitination of RIP1 to inhibit the activation of pro-survival signaling pathways and accelerate the necrosis of MDA-MB-231 cells. The disclosed mechanisms of SKO induced necrosis and apoptosis in our study is firstly reported, and it is believed that SKO could be considered as a potential candidate and further developed for the treatment of triple negative breast cancer.

## Background

Breast cancer accounts for one-tenth of all new diagnosed cancers worldwide every year [1]. It is the most common cancer among women in developing and developed regions and is becoming the leading cause of cancer related death among women worldwide [2]. Breast cancer is presently considered as the most common cancer among Chinese women, which is also occurred in most other countries [3]. Cases from China account for 12.2% of all newly diagnosed breast cancers and 9.6% of global breast cancer deaths [4]. Breast cancer is a complex and diverse disease, and profiles of gene expression contributes a lot for revealing the heterogeneity of it at molecular level [5]. The types of breast cancer range from simple histological types, tumor grade, lymph node status, and predictive markers such as estrogen receptors to more complex classifications based on human epidermal growth factor receptor 2 (HER2) [6]. The classification of breast cancer includes lumen A (Luminal A), lumen B, basal-like, HER2-positive and normal subgroups [7, 8].

Triple negative breast cancer is characterized with no expression of neither estrogen receptor (ER), progesterone receptor (PR) nor HER-2. Hence, it is still a big challenge for clinical treatment, because this type of cancer cell does not respond to hormone therapy or other available targeted drugs [9]. About 15% of patients diagnosed with breast cancer worldwide are classified as triple negative breast cancer [10]. Chemotherapy is still the main strategy for the treatment of triple negative breast cancer. The current clinical intervention agents for triple negative breast cancer mainly include anthracyclines, taxanes, ixabepilone, platinum drugs and biological agents [10]. Recently, EGFR inhibitors have been used as a treatment for triple negative breast cancer [11]. There is currently no proven effective single drug that has a clear susceptibility to triple negative breast cancer [12, 13]. Moreover, the gradual resistance of tumor cells to apoptosis and recurrence are the main factors leading to the failure of triple negative breast cancer treatment [14].

Inactivation or down regulation of pro-apoptotic effectors and up regulation of anti-apoptotic factors are commonly found in advanced cancer cells that resistant to apoptosis. The inhibitor of apoptosis protein (IAP) family, which includes c-IAP1, c-IAP2, XIAP, survivin, livin, and NAIP in humans, consists of an evolutionarily conserved group of apoptosis inhibitors containing a conserved 70 amino acid BIR (baculovirus inhibitor repeat) domain [15]. Overexpression of IAP family members in cancer cell lines and primary tumors suggests an important role for these proteins in cancer progression [16]. cIAP1 and cIAP2, the E3 ubiquitin ligases, play important roles in regulation of tumor necrosis factor (TNF) receptor superfamily [17]. cIAP1 and cIAP2 function through direct interactions to inhibit the activity of several caspases, including caspase-3, caspase-7, and caspase-9 [18]. In addition, binding of IAP family members to the mitochondrial protein Smac blocks their interaction with caspase-9, thereby allowing the processing and activation of the caspase [15]. In addition, the receptor-interacting protein 1 (RIP1), one of the substrate of cIAP1 and cIAP2, can be modified by cIAP1/2 with Lys63-linked poly-ubiquitin through combination with TNF receptor 1 associated death domain (TRADD) or TNF receptor 1 (TNFR1) in TNFR 1 signaling [19, 20]. Ubiquitin the kinase RIP1 finally activates the mitogen-activated protein kinase (MAPK) signaling and the canonical nuclear factor κB (NF-κB) pathway to promote cell survival [19, 20]. To present, it is considered that the initiation of programmed necrosis, ‘necroptosis’, by death receptors (such as tumor necrosis factor receptor 1) requires some key elements, such as activation of RIP1and RIP3, which executes the disintegration of mitochondrial, lysosomal and plasma membranes. Several distinct molecular mechanisms were involved in the execution of TNFR1-initiated necroptosis. Some of these effectors can also be activated by other necroptotic triggers, including pathogens associated molecular patterns (PAMPs) and DNA damage, in which ROS production is not essential for all instances of TNF-induced necrosis [21].

Chinese medicine, a complementary and alternative strategy for treatment kinds of diseases for thousands of years in China, is now also gradually introduced into cancer therapies. Many bioactive phytochemicals derived from Chinese herbal medicine, such as berberine, curcumin, tanshinone IIA, baicalein, resveratrol, dioscin, silibinin, wogonin, quercetin and celastrol, showed potential therapeutic effects with low side effects for cancers [22]. Nowadays, bioactive constituents form Chinese herbal medicine is attracting more and more researchers’ attention for the treatment of cancers. Shikonin (SKO) is a naphthoquinone dye that isolated from the dried root of Chinese herbal medicine *Arnebia euchroma* (Royle) Johnst. or *Arnebia guttata* Bunge, which has been used in traditional Chinese medicine for thousands of years. This herb is traditionally used for promoting blood circulation and removing rashes and spots. It is also used for the treatment of measles impervious, sores, eczema, water and fire scald. Evidences point out that SKO possesses various pharmacological activities, including anti-inflammatory and anti-tumor [23, 24]. Overwhelming evidences demonstrate that SKO exerts its cytotoxicity in cancer cells by multiple signaling pathways in aspects of oxidative stress, DNA damage, glycolysis, cell cycle arrest, apoptosis autophagy as well as necroptosis and so on [24]. It is report that SKO could induce cell cycle arrest and provoke activation of RIP1-RIP3 signaling in MDA-MB-468 cell line accompanied by increase of ROS levels and a reduction in mitochondrial membrane potential [25]. Recent work also demonstrates that SKO reverses epithelial-to-mesenchymal transition (EMT) in MDA-MB-231 and 4T1 cells by inhibiting the activity and downregulation the expression of β-catenin to decrease its nuclear accumulation, binding to T-cell factor consensus oligos, and transcription of EMT-related genes. Moreover, upregulation of glycogen synthase kinase 3β (GSK-3β) levels by SKO facilitates phosphorylation and decreasing levels of β-catenin, which also plays a great role in inhibiting lung metastasis of MDA-MB-231 cells in NOD/SCID mice [26]. Previous results also point out that SKO induces apoptosis in MDA‑MB‑231 and 4T1 cells by activation of p38 signaling pathway [27], and inhibition of matrix metalloproteinase-9 activation to block the migration and invasion of MDA-MB-231 is also discovered [28]. Although many efforts have been exerted to disclose the mechanisms of SKO for the treatment of triple negative breast cancers, it is generally considered that many signaling pathways might contribute to the treatment effects of SKO for breast cancers. In our previous study, it is found SKO triggered ubiquitination and degradation of cIAP1 and cIAP2, which plays a key role in regulation of necrosis and apoptosis in MDA-MB-231 cells. The underlying mechanisms for this pathway is not fully addressed and quite different from that in the previous reports. Based on the novel anti-cancer effects of SKO for triple negative breast cancer *in vitro* and *vivo*, it is necessary to deep explore the potential and additional underlying mechanisms of SKO, and bring benefits for further research and development of this compound for clinical treatment. Hence, in this study, we attempt to disclose the mechanisms of SKO induced necrosis and apoptosis in triple negative breast cancer cells.

## Materials and methods

### Chemicals and reagents

Phosphate-buffered saline (PBS), penicillin-streptomycin, 0.25 %-EDTA and fetal bovine serum (FBS) were purchased from Gibco (Carlsbad, CA, USA). 3-[4,5-dimethylthiazol-2-yl]-2,5-diphenyl-2H-tetrazolium bromide (MTT), Lactacystin (LAC) necrostatin-1 (Nec-1) and SKO (≥ 98 %) were purchased from Sigma-Aldrich (St. Louis, MO, USA). RIPA lysis buffer was purchased from Beyotime Biotechnology Company (Shanghai, China). Protein A/G agarose was purchased from Santa Cruz Biotechnology (Santa Cruz, CA, USA). Primary antibodies, including PARP, GAPDH, receptor interacting protein 3 kinase (RIP3), phospho-RIP3 (Ser227), RIP1, phospho-RIP1 (Ser166), RIP3, phospho-MLKL (Ser358), cIAP1, cIAP2, XIAP, FADD, DR5, DR4, phospho-IKK_α/β_, and ubiquitination were obtained from Cell Signaling Technology (Danvers, MA, USA). All secondary antibodies were also purchased from Cell Signaling Technology (Danvers, MA, USA). Enzyme linked immunosorbent assay (ELISA) kit for determination of human TNF-α content in medium was purchased from Biolegend (San Diego, CA, USA). Annexin V/propidium iodide (PI) assay kit was purchased from BD Biosciences (Qume Drive San Jose, CA, USA). TRIzol reagent for extraction of total RNA from cells was purchased from Invitrogen® Thermo Fisher Scientific Inc. (Waltham, MA, USA). PrimeScript™ RT Reagent Kit with gDNA Eraser for cDNA synthesis and TB Green® Premix Ex TaqTM II (Tli RNase H Plus) for real-time PCR analysis were from TakaRa Bio Inc. (Nojihigashi Kusatsu, Gumma, Japan).

### Cell culture

Human breast cancer cell line MDA-MB-231 was purchased from American Type Culture Collection (Rockville, MD, USA) and was routinely cultured in RPMI 1640 medium supplemented with 10 % FBS, 100 U/mL penicillin, and 100 µg/mL streptomycin in a humidified incubator under 95% air and 5% CO_2_ at 37 ^o^C.

### Cell viability assay

MDA-MB-231cells were seeded in 96-well plates at a density of 5 × 10^3^ cells/well. When reaching approximately 70–80% confluence, cells were treated with DMSO as blank control, or different concentrations of SKO. Then cell viability was determined by incubation with medium containing MTT (1 mg/mL) for 4 h, followed by dissolving the formazan crystals with DMSO. The absorbance at 570 nm was determined by a microplate reader (SpectraMax M5, Molecular Devices, USA) and presented as relative cell viability. The results were analyzed based on at least three independent experiments.

### Flow cytometry analysis

MDA-MB-231 cells were seeded in 6-well plates at a density of 2 × 10^5^ cells/well and grown for 24 h. Cells were treated with or without different concentrations of SKO for indicated time, and then culture medium in each well were collected and centrifuged (300 g, 5 min) to harvest the floating cells. Subsequently, 100 µL/well 0.25 % trypsine (without phenol red, calcium and magnesium) were added and incubated at 37 ^o^C for 5 min for detaching cells in the plate, followed by adding 1 mL culture medium and centrifuging to get the cells. The trypsinized and floated cells in each well were respectively mixed together and washed with PBS for twice, then diluted by PBS to 1 × 10^6^ cells/mL followed by 15 min of incubation with Annexin V and PI staining in the dark at room temperature. Loss of cell membrane integrity was assessed by the annexin V-fluorescein (FITC)/propidium iodide (PI) double staining assay. The cell suspensions were counted using FACScan flow cytometer (Becton Dickinson, Franklin Lakes, NJ, USA), 1 × 10^4^ events per run were recorded for each sample. The excitation and emission wavelengths for annexin V-FITC detection were set at 488 and 525 nm, respectively, and PI were set at 575 and 615 nm. The results were analyzed based on at least three independent experiments.

### Measurement of TNF-α content in medium

MDA-MB-231cells were seeded in 12-well plates at a density of 5 × 10^3^ cells/well. Cells were treated with or without different concentrations of SKO for indicated time when cells reaching approximately 70–80% confluence in each well. Then cell culture medium was collected and centrifuged for determination content of human TNF-α level in the culture medium according to the manufacturer’s instructions.

### Real time- quantitative PCR analysis of targeted genes

MDA-MB-231 cells were seeded in 6-well plates at a density of 2 × 10^5^ cells/well and grown for 24 h. Cells were treated with or without different concentrations of SKO for 1 h, and total RNA from treated cells were extracted using TRIzol reagent according to the manufacturer’s instructions. Then 2 µg RNA from each sample and PrimeScript™ RT Reagent Kit with gDNA Eraser were used for cDNA synthesis. TB Green® Premix Ex TaqTM II (Tli RNase H Plus) was used for real-time PCR analysis performed on Applied Biosystems (Thermo Fisher Scientific, Waltham, MA, USA). The following primers were used: F 5′-TCCTTCAGACACCCTCAAC-3′, R 5′-CAGGGATCAAAGCTGTAGGC-3′ for *TNF-α*, F 5′-GAAATCCCATCACCATCTTCCAGG-3′, R 5′-GAGCCCCAGCCTTCTCCATG-3′ for *GAPDH*. The transcription of targeted gene was normalized to *GAPDH*, and relative transcription of targeted gene was calculated by 2 ^−ΔΔ Ct^.

### Western blot analysis

MDA-MB-231cells were seeded in 100 mm dish at a density of 1 × 10^6^ cells/dish. Cells were treated with or without different concentrations of SKO for indicated time when cells reaching approximately 70–80 % confluence in each dish. Then cells were harvested and cell lysates were prepared in RIPA buffer (containing 1 mM PMSF and 1 mM phosphatase inhibitor) on ice. The total protein concentrations were determined by BCA Protein Assay Kit (Pierce Biotechnology). Equal amounts of protein for each sample were separated by SDS-PAGE and transferred to PVDF membranes. The membranes were blocked with 5 % nonfat milk (dissolved in TBST buffer) for 3 h at room temperature and immunoblotted with polyclonal primary antibodies. After washed with TBST for three times, the immunoblots were incubated with peroxidase-conjugated secondary antibodies (diluted at 1:1000) at room temperature for 3 h. The proteins were detected with SuperSignal™ West Femto Maximum Sensitivity Substrate (Thermo Fisher Scientific, Waltham, MA, USA).

### Immunoprecipitation

To examine interactions between proteins in cells treated with or without SKO, the IP/Western blot analyses were performed. The detailed procedure is described as following: the cellular protein was extracted and precleared by incubation with protein A/G agarose beads at 4 °C for 1 h. Next, the samples were incubated with the beads coupled to the targeted primary antibodies at 4 °C overnight. The immune complex was washed with RIPA lysis buffer for three times and boiled in protein loading buffer for 5 min at 95 ^o^C. Finally, the immunoprecipitate was separated with SDS-PAGE and transferred to PVDF membranes.

### Statistical analysis

Data were expressed as mean ± SEM based on at least three independent experiments and analyzed on Graphpad Prism 6 (GraphPad Software, San Diego, CA, USA). The significance of differences between groups was assessed by one-way analyses of variance (ANOVA) using SPSS software 16.0 (Chicago, IL, USA). *p* < 0.05 and *p* < 0.01 were considered as significant difference and very significant difference, respectively.

## Results

### The cytotoxicity of SKO in cells was dose and time dependent and might be independent of activation of RIP1/MLKL/RIP3 axis

**Fig. 1:**
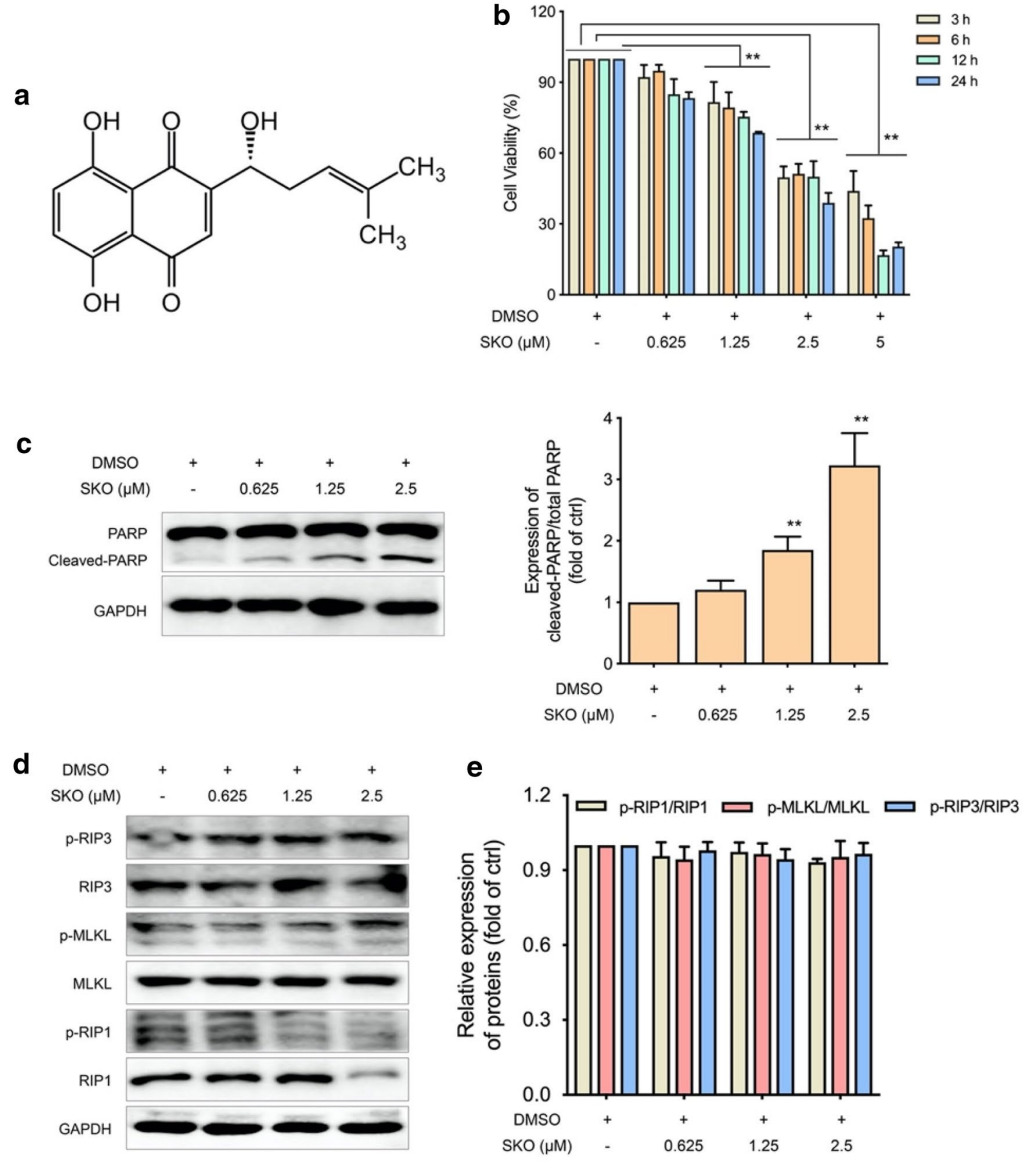
SKO showed cytotoxicity against MDA-MB-231 cells in a concentration- and time-dependent manner that independent of activation of RIP1/MLKL/RIP3 axis. **a** Chemical structure of SKO. **b** Cell viability of MDA-MB-231 cells treatment with different concentrations of SKO for different time. **c** The expression and quantitatively analysis of PARP and its cleaved product in cells treated with SKO for 12 h. **d** The expression of RIP1, RIP3, MLKL and their phosphorylated proteins in cells treated with SKO for 12 h. **e** Quantitatively analysis the relative expression of phosphorylated RIP1, RIP3, MLKL to total protein. Data was represented as means ± SEM. **p < 0.01 vs. untreated control group

SKO, a naphthoquinone dye, is one of the most important constituents in the root of Lithospermum (*Zicao* in Chinese), which is from the family of *Arnebia euchroma* (Royle) Johnst. or *Arnebia guttata* Bunge. The chemical structure of SKO is shown in Fig. 1a. To assess the cytotoxicity of SKO on MDA-MB-231 cells, the anti-proliferative effect of SKO with different concentration and treatment time using MTT assay method was examined. As shown in Fig. 1b, the cytotoxicity of SKO on MDA-MB-231 cells was not obvious at concentration of 0.625 µM, and the cytotoxicity of SKO was significantly increased when the concentration increased from 1.25 to 5 µM. It was also found that the cytotoxicity of SKO was significantly increased when prolonging treatment time with cells. The relative viability of MDA-MB-231 cells treatment with 1.25 µM SKO was 81.6% for 3 h, and the relative viability of was decreased to 68.7% treatment with the same concentration of SKO for 12 h. Meanwhile, it was found the relative viability of MDA-MB-231 cells treatment with 5 µM SKO was 44.0% for 3 h, and cells almost lost their viability treatment with 5 µM SKO for 12 and 24 h. The results demonstrated that SKO induced MDA-MB-231 cell death in both concentration and time dependent manners.

Then western blot analysis was performed to investigate on death related proteins in cells treated with SKO. As shown in Fig. 1c, the cleaved product of PARP was significantly increased when cells treated with 0.625–2.5 µM SKO for 12 h. Quantitative results showed that the relative expression of cleaved PARP to total PARP was 1.9- and 3.2-folds to that of control in cells treated with 1.25 and 2.5 µM SKO for 12 h, respectively. To further investigate on the role of RIP1, RIP3 as well as MLKL for SKO induced cytotoxicity in cells, the expression of these proteins and corresponding phosphorylated products were also detected. As shown in Fig. 1d, e, the expression of RIP3 and MLKL in cells treated with 0.625–2.5 µM SKO for 12 h were not significantly changed. The expression of RIP1 was significantly decreased when cells treated with 2.5 µM SKO for 12 h. However, quantitatively analysis for the relative expression of phosphorylated RIP1, RIP3 as well as MLKL to that of corresponding total protein were not significantly changed when cells treated with 2.5 µM SKO for 12 h. Hence, it is deduced that SKO induced cytotoxicity in MDA-MB-231 cells might be independent of activation of RIP1/RIP3/MLKL axis. Taken all these results together, it was considered that SKO induced cytotoxicity in MDA-MB-231 cells in both concentration- and time-dependent manners, and RIP1/RIP3/MLKL axis was not involved in SKO induced cell death in MDA-MB-231 cells.

#### SKO induced cells both necrosis and apoptosis in a concentration- and time-dependent manner

**Fig. 2:**
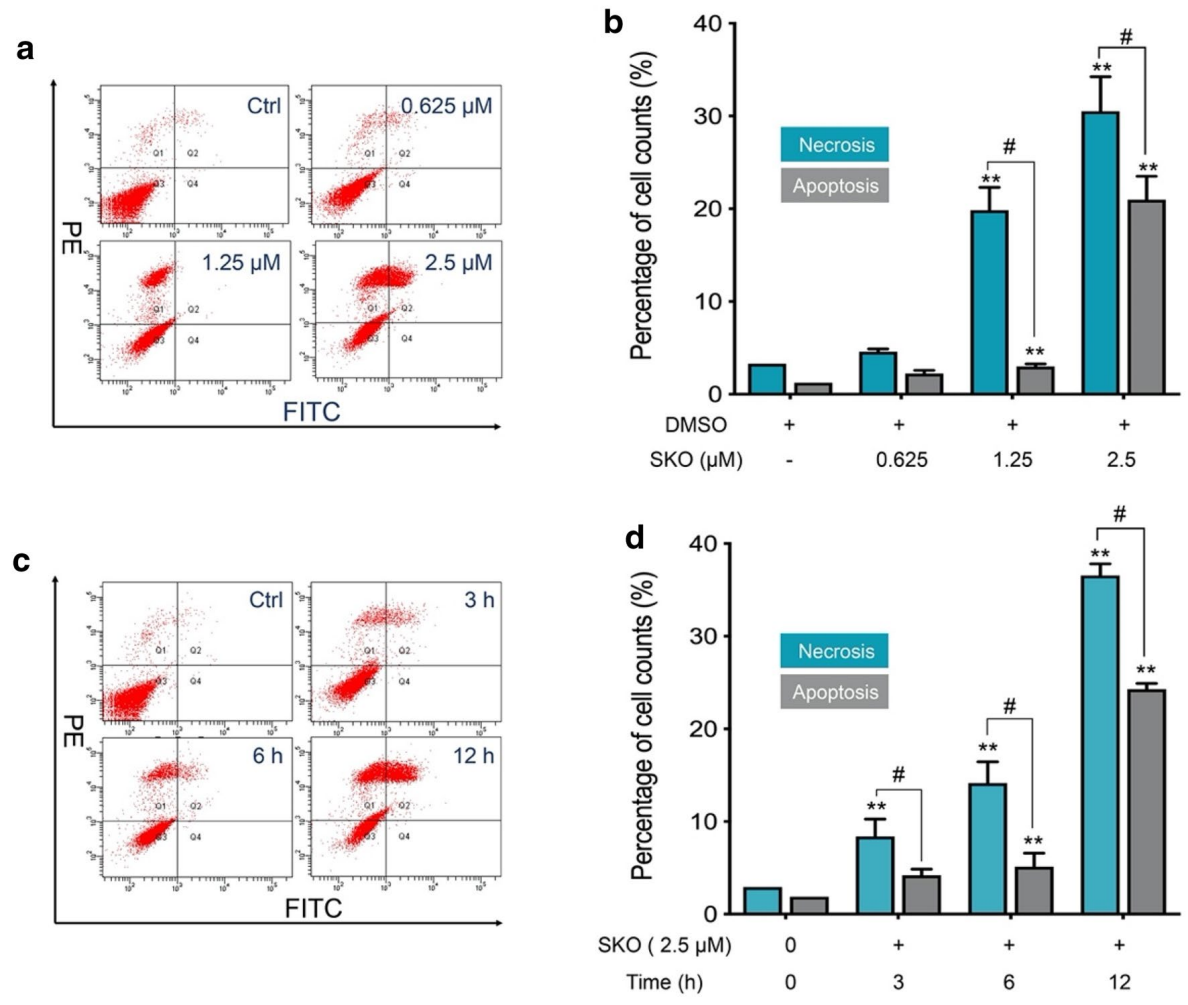
SKO induced cell necrosis and apoptosis both in a concentration- and time-dependent manner. **a** Cell count for necrotic and apoptotic MDA-MB-231 cells treated with or without different concentration of SKO. **b** Statistical analysis of necrotic and apoptotic MDA-MB-231 cells treated with or without different concentration of SKO. **c** Cell count for necrotic and apoptotic MDA-MB-231 cells treated with 2.5 µM SKO for different treatment time. **d** Statistical analysis of necrotic and apoptotic MDA-MB-231 cells treated with 2.5 µM SKO for different treatment time. Data was represented as means ± SEM. **p < 0.01 vs. untreated control group. ^#^*p <* 0.01 compared with the corresponding group

To further explore the characteristics of SKO induced cell death in MDA-MB-231 cells, flow cytometry was used for counting the necrotic and apoptotic cells after treatment with SKO. As shown in Fig. 2a, cells treated with 1.25 and 2.5 µM SKO for 12 h mainly induced both necrosis and apoptosis in cells. However, statistical analysis demonstrated that cells treated with 1.25 and 2.5 µM SKO for 12 h significantly induced cell necrosis. As shown in Fig. 2b, cells treated with 1.25 µM SKO for 12 h induced 19.9% cell necrosis and 3.0% cells apoptosis. When cells treated with 2.5 µM SKO for 12 h, 30.5% necrotic and 21% apoptotic cells were detected, respectively. Results demonstrated that the number of apoptotic cells was obviously increased with increasing of dosage for cells from 1.25 to 2.5 µM, and the number of necrotic cells was increased in a slighter extent. To understand the effect of treatment time for cell necrosisis and apoptosis, experiments were performed to count the number of necrotic and apoptotic cells treated with 2.5 µM SKO for different treatment times. As shown in Fig. 2c, cells treated with 2.5 µM SKO for 3–12 h mainly induced both necrosisis and apoptosis in cells. Cell count results showed that cells treated with 2.5 µM SKO for 3, 6 and 12 h resulted in 8.4%, 14.1% and 36.2% necrotic cells, respectively. Meanwhile, it was found that the number of apoptotic cells was rapidly increased from 5.1 to 24.3% when cells treated with 2.5 µM SKO for 6 and 12 h. The manner of cell death induced by SKO was mainly composed of necrosisis, and the ratio of apoptotic cells was obviously increased with prolonged treatment time. Taken these results together it was found that SKO induced cells both necrosis and apoptosis both in a concentration- and time-dependent manner, and SKO induced cell death was mainly composed of cell necrosisis at low dosage (1.25 µM) and short treatment time (6 h). The ratio of apoptotic cells was significantly increased with increased dosage (2.5 µM) and prolonged treatment time (12 h).

#### SKO induced degradation of RIP1, cIAP1 and cIAP2 in cells

**Fig. 3:**
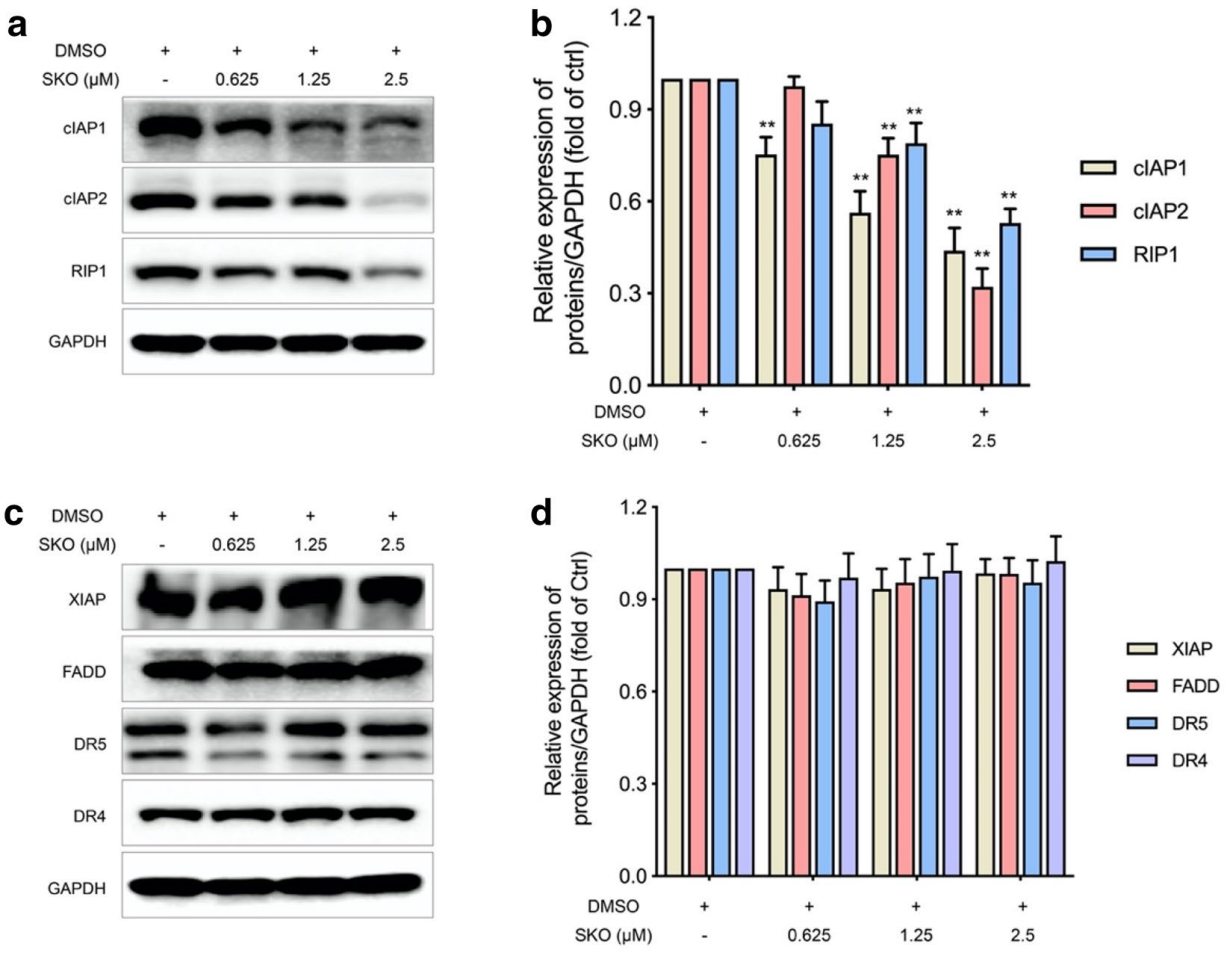
SKO induced degradation of cIAP1, cIAP2 and RIP1 in MDA-MB-231 cells. **a** The expression of cIAP1, cIAP2 and RIP1 in cells treated with SKO at the indicated concentrations for 12 h. **b** Semi-quantitative the expression of cIAP1, cIAP2 and RIP1 in cells treated with SKO at the indicated concentrations for 12 h. **c** The expression of XIAP, FADD, DR5 and DR4 in cells treated with SKO at the indicated concentrations for 12 h. **d** Semi-quantitative the expression of XIAP, FADD, DR5 and DR4 in cells treated with SKO at the indicated concentrations for 12 h. Data was represented as means ± SEM. **p < 0.01 vs. untreated control group

To better understand the underlying mechanisms of SKO induced necrosis and apoptosis in cells, key proteins may be involved in regulation of the process by SKO were detected. As shown in Fig. 3a, the expression of cIAP1, cIAP2 as well as RIP1 were significantly decreased in a dose dependent manner in MDA-MB-231 cells. Semi-quantitative analysis demonstrated that the expression of cIAP1 in cells treated with 0.625–2.5 µM SKO for 12 h was significantly decreased to 0.75, 0.56 and 0.44-fold of that in untreated cells (Fig. 3b), respectively. The expression of cIAP2 in cells at concentration of 1.25–2.5 µM SKO was significantly decreased to 0.75- and 0.32-fold of that in untreated cells, respectively. At the same time, it was also found that the expression of RIP1 was significantly decreased to 0.79- and 0.53-fold of that in untreated cells, respectively. The expression of other proteins and receptors in cells which played key roles in regulation necrosis and apoptosis, such as XIAP, FADD, DR5 and DR4, were simultaneously detected. Results demonstrated that the expression of these key proteins in cells were not obviously changed treated with 0.625–2.5 µM SKO for 12 h (Fig. 3c). Semi-quantitative analysis also found the expression of these proteins were not significantly affected by treatment with SKO at the effective concentration (Fig. 3d). Taken these results together, it is deduced that some key regulators for cell necrosisis and apoptosis, such as XIAP, FADD, DR5 and DR4, might not be involved in the process of SKO induced cytotoxicity in MDA-MB-231 cells. And results indicated the significantly decreased expression of cIAP1, cIAP2 and RIP1 in cells treated with SKO might play great role in SKO induced cell death, which should be further uncovered.

#### SKO facilitated ubiquitination of cIAP1 and cIAP2 in triple negative breast cancer cells

**Fig. 4:**
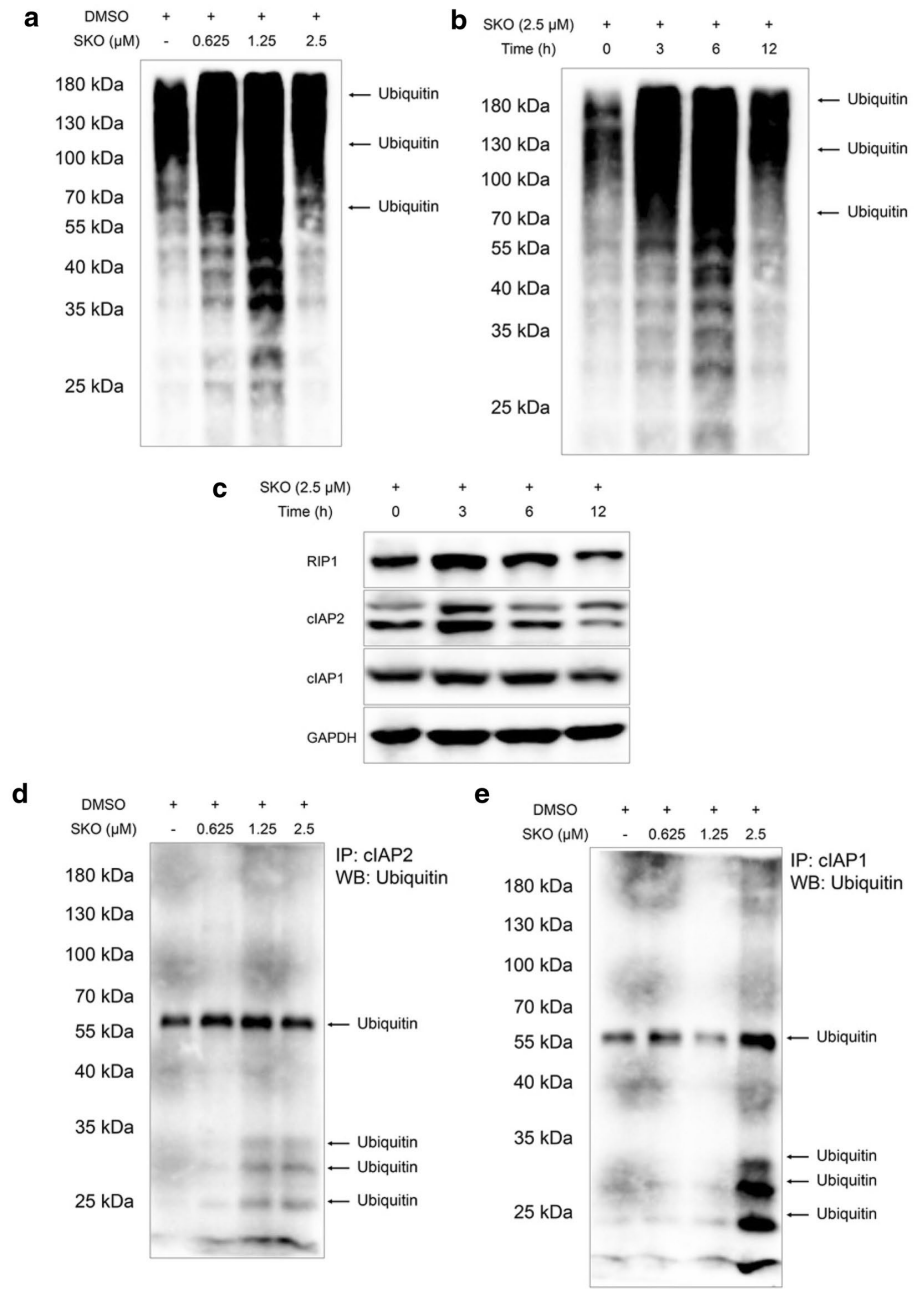
SKO facilitated ubiquitination of cIAP1 and cIAP2 in MDA-MB-231 cells. **a** Ubiquitination of proteins in cells treatment with or without of SKO at the indicated concentrations for 12 h. **b** Ubiquitination of proteins in cells treatment with SKO for the indicated time. **c** The expression of cIAP1, cIAP2 and RIP1 in cells treated with SKO for the indicated time. **d** Immunoprecipitation assay for ubiquitination of cIAP2 in cells treatment with or without of SKO at the indicated concentrations for 12 h. **e** Immunoprecipitation assay for ubiquitination of cIAP1 in cells treatment with or without of SKO at the indicated concentrations for 12 h

Since cIAP1 and cIAP2 play key roles in auto-ubiquitination and ubiquitination of other proteins, the ubiquitination of proteins in cells were detected to determine whether ubiquitination of cIAP1 and cIAP2 induced by SKO in cells facilitated degradation of cIAP1 and cIAP2, which further contributes to the cytotoxicity of SKO for MDA-MB-231 cells. As shown in Fig. 4a, ubiquitination of proteins was widely found in cells treated with 0.625-2.5 µM SKO for 12 h. It was also found cells treated with 1.25 µM SKO for 12 h induced the most obvious ubiquitination of proteins compared with that of cells treated with 0.625 and 2.5 µM SKO for 12 h. The effects of treatment time on ubiquitination of proteins in cells was simultaneously detected, and results demonstrated the ubiquitination level for proteins in cells was obviously increased with prolonged treatment time (Fig. 4b). Among all the results from ubiquitination of proteins in cells with different treatment times, it was found cells treated with 2.5 µM SKO for 6 h induced the maximum extent of ubiquitination of proteins. Subsequently, the expression of cIAP1, cIAP2 and RIP1 in cells with  indicated treatment time at the indicated concentration were detected, it was found that the expression of these proteins in cells were gradually decreased with prolonged of treatment time (Fig. 4c). Hence, it is deduced that the cytotoxicity of SKO in MDA-MB-231 cells might be correlated with the expression of cIAP1, cIAP2 and RIP1. To further determine the ubiquitination of cIAP1 and cIAP2 induced by SKO, immunoprecipitation experiments were performed to specifically detect the ubiquitination of these two proteins. As shown in Fig. 4d, the ubiquitination of cIAP2 were obviously increased accompanied with increase dosage of SKO for cells. Meanwhile, it was also found that the induction of ubiquitination of cIAP1 in cells treatment with SKO was increased, especially for that of cells treatment with 2.5 µM SKO for 12 h. Taken these results together, it was deduced that the ubiquitination of proteins induced by SKO was widely observed and might played an important role for the cytotoxicity of SKO in MDA-MB-231 cells. At the same time, it was considered that ubiquitination of cIAP1 and cIAP2 induced by SKO might also play a great role in regulation of necrosisis and apoptosis of cells.

#### SKO promoted ubiquitination and proteasome degradation of cIAP1 and cIAP2, and interfered of RIP1 pro-survival signaling

**Fig. 5:**
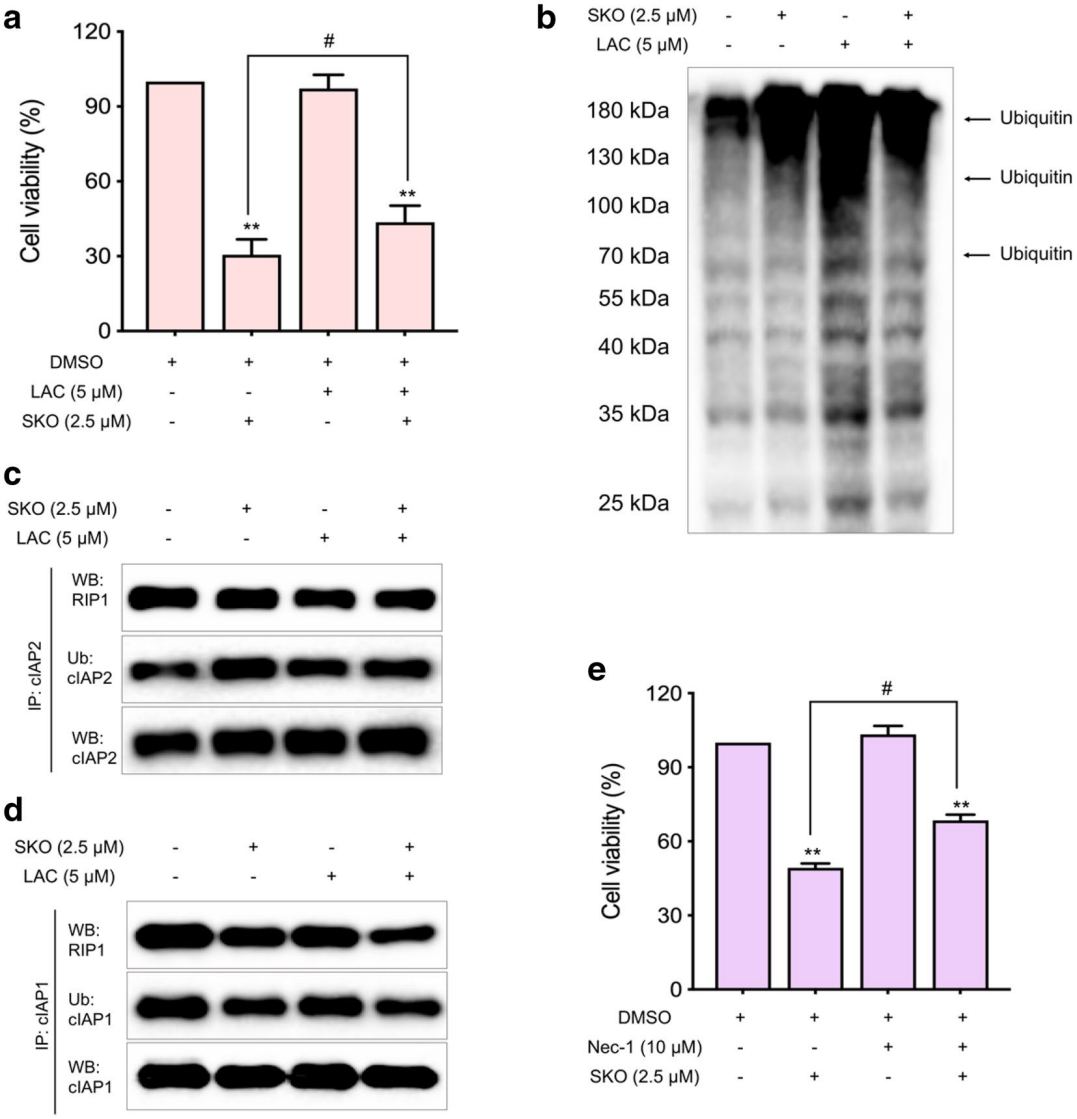
SKO promoted ubiquitination and proteasome degradation of cIAP1/cIAP2 and interfered RIP1 prosurvival signaling. **a** The relative viability of MDA-MB-231 cells treated with or without 5 µM LAC and 2.5 µM SKO. **b** The ubiquitination of proteins in cells treated with or without 5 µM LAC and 2.5 µM SKO. **c** Immunoprecipitation assay for detecting ubiquitination of cIAP2 and interaction with RIP1 in cells treated with or without 5 µM LAC and 2.5 µM SKO. **d** Immunoprecipitation assay for detecting ubiquitination of cIAP1 and interaction with RIP1 in cells treated with or without 5 µM LAC and 2.5 µM SKO. **e** The relative viability of cells treated with or without 10 µM Nec-1 and 2.5 µM SKO. Data was represented as means ± SEM. **p < 0.01 vs. untreated control group. ^#^*p <* 0.01 compared with the corresponding group

To validate the role of ubiquitination of cIAP1 and cIAP2 in SKO induced toxicity in MDA-MB-231 cells, the ubiquitination-proteasome inhibitor, LAC, was used. As shown in Fig. 5a, it was found that the relative cell viability was 30.6% when cells were treated with 2.5 µM SKO for 12 h. The relative cell viability was significantly increased to 43.6% by addition of 5 µM LAC, which showed no obvious cell toxicity at the same concentration for the indicated treatment time. Meanwhile, it was also observed that the ubiquitination of proteins in cells treated with 5 µM LAC and 2.5 µM SKO for 12 h was increased compared with that of only treatment with SKO (Fig. 5b). The results demonstrated that proteasome degradation of proteins dependent on ubiquitination played crucial roles for SKO in regulation of cell necrosis and apoptosis. Immunoprecipitation experiments found that the ubiquitination level of cIAP2 was slightly decreased in cells treated with 5 µM LAC and 2.5 µM SKO for 12 h compared with that of treatment with SKO (Fig. 5c). The same results were also found for the ubiquitination level of cIAP1 in cells treated with 5 µM LAC and 2.5 µM SKO for 12 h compared with that of treatment with SKO (Fig. 5d). Additionally, it was found that the ubiquitination level of RIP1 was also decreased in cells treated with 5 µM LAC and 2.5 µM SKO for 12 h compared with that of treatment with SKO (Fig. 5c, d), which was accompanied with decreased interactions with cIAP1 and cIAP2 in cells treated with 5 µM LAC and 2.5 µM SKO for 12 h compared with that of treatment with SKO. Hence, it was deduced ubiquitination and activation of RIP1 through interacting with cIAP1 and cIAP2 played a key role in regulation of cell necrosis and apoptosis. Subsequently, the inhibitor of RIP1, Nec-1, was used for validating the regulation role of RIP1 for SKO induced cytotoxicity in cells. As shown in Fig. 5e, it was found that Nec-1 significantly rescued the cytotoxicity of SKO. The relative cell viability was 49.2% when cells treated with 2.5 µM SKO for 12 h, and the relative cell viability was significantly increased to 68.5% by addition of 10 µM Nec-1. These results demonstrated that cells treatment with SKO lead to increase auto-ubiquitination and degradation of cIAP1 and cIAP2, and interfered ubiquitination and activation of RIP1 in cells, which played a crucial role in regulation of cell necrosis and apoptosis depended on RIP1 activity.

#### SKO treatment significantly increased the auto-secretion of TNF-α to induce cell apoptosis and necrosis

**Fig. 6:**
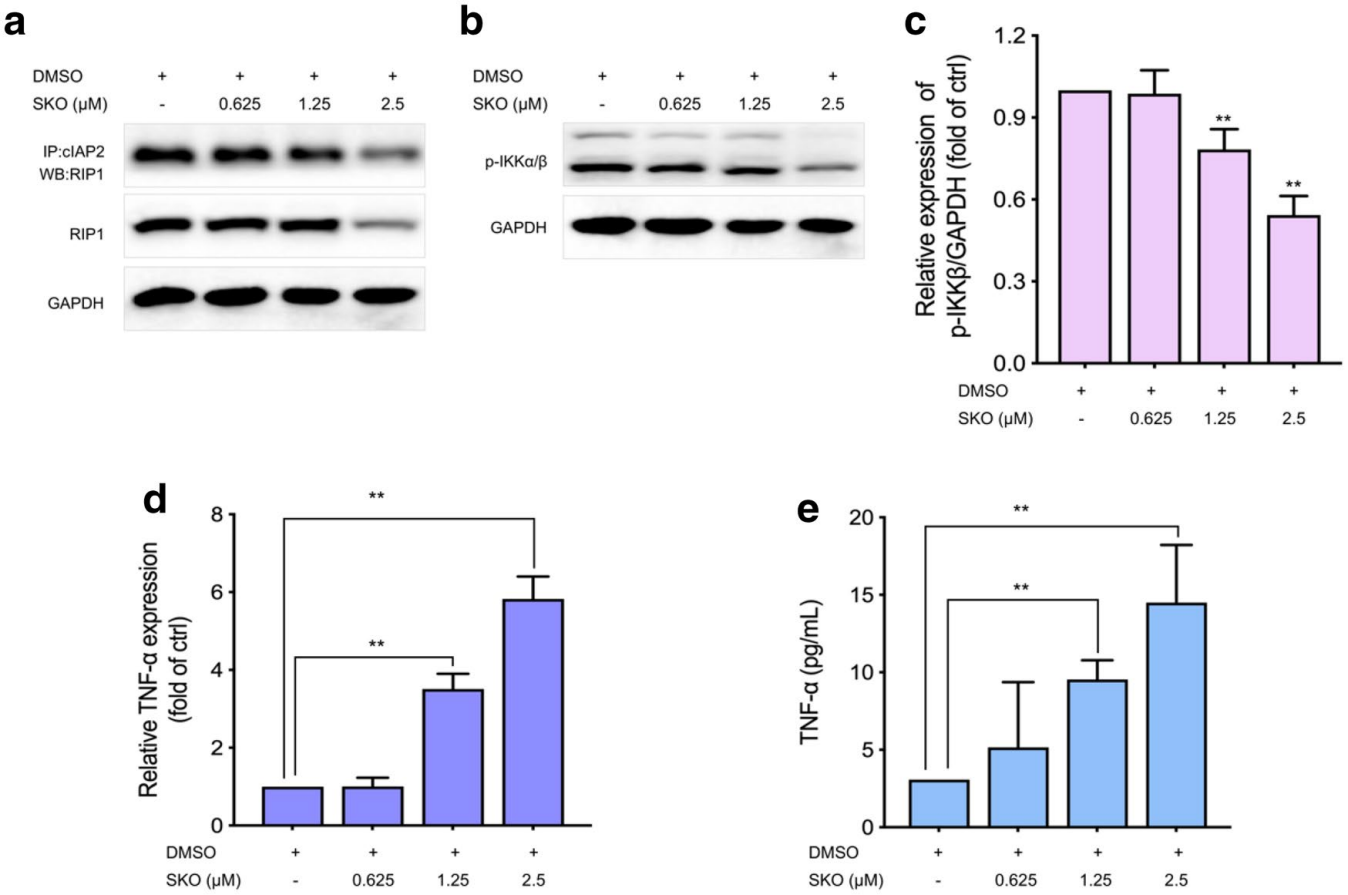
SKO treatment significantly increased the auto-secretion of TNF-α in MDA-MB-231 cells to induce cell apoptosis and necrosis. **a** Immunoprecipitation assay of the interactions between RIP1 and cIAP2. **b** The expression of phosphorylation IKK_α/β_ in cells treatment with or without of SKO at the indicated concentrations. **c** Semi-quantitation the expression of phosphorylation IKK_α/β_ in cells treatment with or without of SKO at the indicated concentrations. **d** The gene expression of *TNF-α* in cells treated with or without of SKO at the indicated concentrations. **e** The concentration of TNF-α in culture medium. Data was represented as means ± SEM. **p < 0.01 vs. untreated control group

To further understand the role and corresponding mechanisms of RIP1 in SKO induced cytotoxicity in cells, immunoprecipitation assay was performed to determine the interactions between cIAP2 and RIP1. As shown in Fig. 6a, it was found that the relative amount of RIP1 interacted with cIAP2 in cells treatment with different concentrations of SKO was decreased. Meanwhile, the phosphorylated products of IKK_α/β_ was gradually decreased in cells treatment with different concentrations of SKO (Fig. 6b). The relative expression of phosphorylation IKK_α/β_ were significantly decreased to 78.3 % and 54.3 % in cells treated with 1.25 and 2.5 µM SKO compared with that of untreated cells, respectively (Fig. 6c). Then it was questioned whether the activation of IKK_α/β_ was involved in the expression and release of TNF-α in cells treated with SKO. As shown in Fig. 6d, the gene expression of *TNF-α* in cells was significantly increased by SKO stimulation. It was found that the relative gene expression of *TNF-α* in cells treated with 1.25 and 2.5 µM SKO for 1 h were significantly increased by 3.5- and 5.8-folds to that of untreated cells, respectively. Quantitative determination of the release of TNF-α in culture medium was also performed, and results found that treatment with SKO facilitated auto-secretion of TNF-α, and the release of TNF-α stimulated by SKO was in a concentration-dependent manner. As shown in Fig. 6e, the concentration of TNF-α in culture medium were 9.5 and 14.5 pg/mL when cells treated with 1.25 and 2.5 µM SKO for 12 h, respectively, which was significant higher that of in control group (3.1 pg/mL). Taken these results together, SKO treatment might interfere the signaling interactions between cIAPs and RIP1, as well as facilitated the expression and autocrine of TNF-α, which played a crucial role in regulation of cell necrosis and apoptosis depended on TNF-α stimulation.

## Discussion

**Fig. 7:**
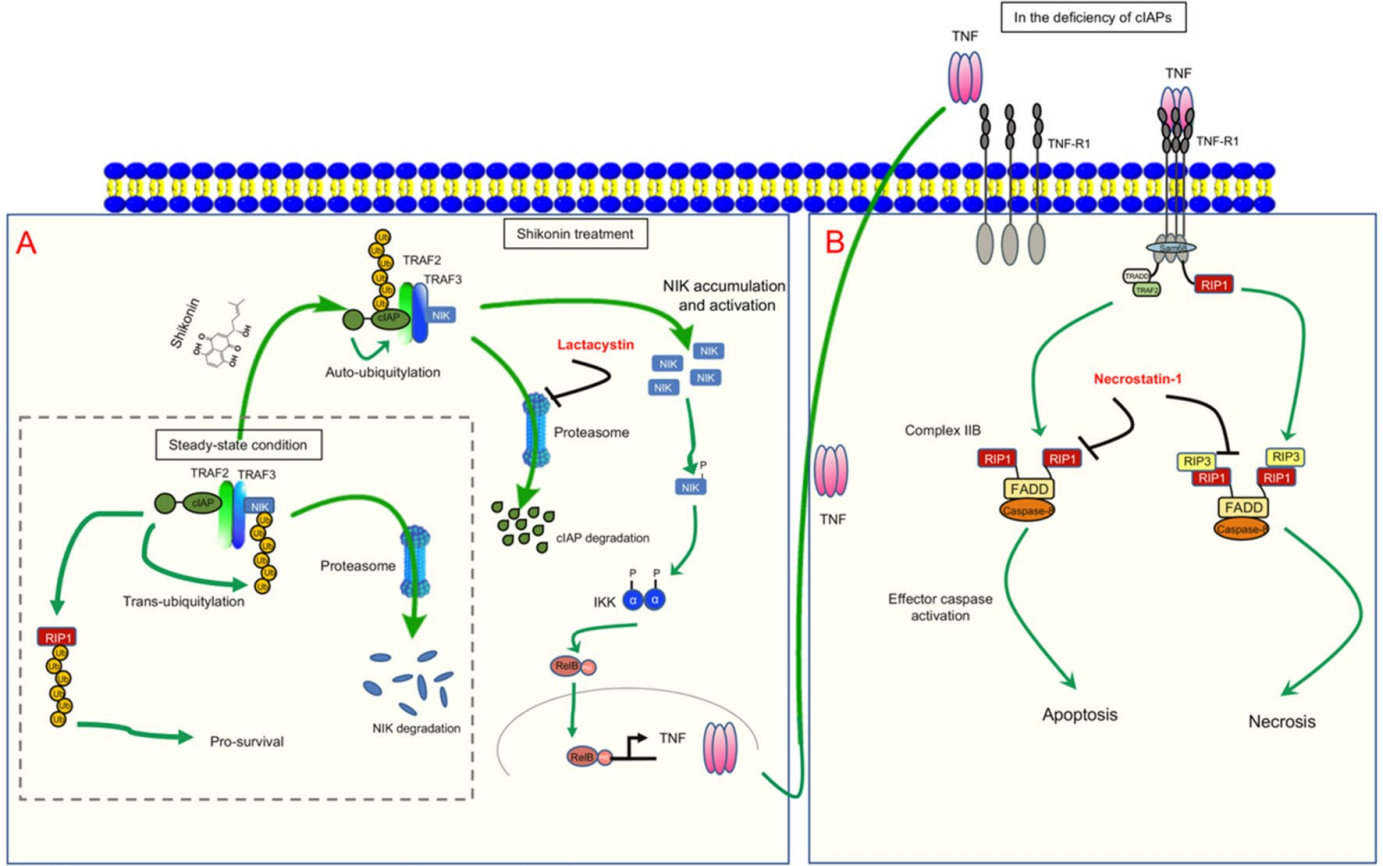
Scheme of potential mechanisms of SKO induced necrosis and apoptosis in MDA-MB-231 cells. **a** In steady-state condition, cIPAs facilitates trans-ubiquitination of NF-κB-inducing kinase (NIK) and degraded by proteasome. Meanwhile, cIPAs play as E3 like ubiquitination enzyme to continuously ubiquitination of RIP1 to promote cell survival. When stimulated by SKO, cIPAs enter into auto-ubiquitination process and degraded by proteasome, which triggers the activation of NIK-TNF signaling to promote the autocrine of TNF in the culture medium. The activity of proteasome is inhibited by proteasome inhibitor LAC. **b** Over accumulation of autocrine TNF binds to the receptor of TNF-R1, and further triggers the TNF induced apoptotic and necrotic signaling pathways. Inhibition the activity of RIP1 by adding Nec-1 successfully rescue cells from SKO induced apoptosis and necrosis

Necroptosis is a kind of regulated cell death mediated by RIP1. RIP1 plays a key role in regulation of cell survival, inflammation and apoptosis, and is identified as a major regulator for necrotic apoptosis. Most of the current understanding of the molecular mechanisms of necrotic apoptosis originated from the study of TNF-α-induced necrotic signaling pathway. TNF-α, an inflammation-related cytokine, plays an important role in inflammation, which itself can not only induce inflammation, but also induce cell apoptosis or necrotic apoptosis under different pathophysiological conditions [29]. In this experiment, it is found that SKO is active in the activation of RIP1 and promotes the auto-secretion of TNF-α in cells, which facilitates cell apoptosis and necrosis (Fig. 6d, e). Stimulating with TNF-α, this cytokine binds to TNFR1 on the surface of the cell membrane, which promotes the recruitment of TNF receptor associated death domain (TRADD) and RIP1 to form a complex with change of TNFR1 conformation. Subsequently, TNF receptor associated factor 2 (TRAF2) and cIAP1/2 are recruited by TRADD and RIP1 to form the Complex I containing with TRADD, RIP1, TRAF2 as well as cIAP1/2 on the cellular membrane. Complex I plays an important role in activating the NF-κB signaling pathway, and thereby promoting cell survival. Activation of NF-κB signaling pathway induces the expression of various prosurvival genes and various anti-apoptotic genes including IAPs, such as cIAP1/2 and cFLIP [30]. In previous studies, NF-κB is considered as an important mediator for many chronic diseases including cancer, asthma, rheumatoid arthritis, diabetes, inflammation, and neurological disorders [31, 32]. In TNF induced NF-κB activation, quinone oxidoreductase 1 (NQO1) plays a pivotal role in activation of NF-κB signaling, and inhibition of NQO1 activity hampers the proliferation, survival, invasion, and metastasis of tumor cells [33]. In our study, it is firstly reported that SKO treatment increased the autocrine of TNF-α in cells, further studies might be performed to investigate on whether activation of NQO1 and NF-κB signaling is involved in SKO induced cell death. In this study, we demonstrated that SKO could promote the cellular auto-ubiquitination and proteasome degradation of cIAP1 and cIAP2 (Fig. 4), which might impair the activation of NF-κB signaling pathway and hence facilitate the cytotoxicity of SKO. The potential mechanisms of SKO induced necrosis and apoptosis in MDA-MB-231 cells is briefly summarized in Fig. 7.

Necroptosis is completely different from cell apoptosis. Inducing tumor into necrotic apoptosis can overcome many obstacles caused by cell apoptosis to achieve tumor therapeutic effects. Necroptosis is cell death that occurs when caspase activity is blocked. When apoptosis is blocked, RIP1 will be activated, thereby activating death receptor ligands such as TNF-α and Fas and causing tumor cell necroptosis [21]. Under the stimulation of TNF, complex I mediates NF-κB activation and MAPK signal transduction, enabling cell inflammation and survival. In the case of cIAP depletion, cytoplasmic complex II initiates FADD-caspase-8-mediated cell death, a process that does not depend on RIP1 activity (complex IIa or IIb) [34].

Studies have shown that RIPK3 activated by phosphorylation can interact with Pellino E3 Ubiquitin Protein Ligase 1 (PELI1) through its FHA domain. PELI1 is a regulatory factor that can target the K363 site KRI of RIPK3 and linked to the polymerized ubiquitin chain leading to its proteasome-dependent degradation, while the phosphorylation of T182 site of RIPK3 is responsible for the activity of RIPK3 kinase and the recruitment of PELI1. PELI1 may provide a steady-state mechanism by promoting the degradation of phosphorylated RIPK3, while preventing abnormal cell death and minimizing the occurrence of necrotic apoptosis [35]. In this experiment, the expression of RIP3, MLKL as well as their corresponding phosphorylated products were not significantly changed (Fig. 1d, e), which demonstrated that activation of RIP3 was not involved in SKO induced cell necrosis and apoptosis. However, it is found that the expression of RIP1 in cells was significantly decreased, which is considered as one of key factors involved in SKO induced cell necrosis and apoptosis. The receptor-interacting protein (RIP) family of serine-threonine kinases (RIP, RIP2, RIP3, and RIP4) are considered as important regulators of cellular stress that trigger pro-survival and inflammatory responses through the activation of NF-κB, as well as pro-apoptotic pathways [36]. In this experiment, it is thought that in addition to the kinase domain, RIP contains a death domain responsible for interaction with the death domain receptor Fas and recruitment to TNF-R1 through interaction with TRADD [37, 38]. RIP-deficient cells show a failure in TNF-mediated NF-κB activation, making the cells more sensitive to apoptosis [39, 40].

Chinese medicine, a great treasure for the treatment of various kinds of diseases, is now gradually used as adjunctive therapy for improving the survival of cancer patients as well as multi-drug resistance for many cancers [41–43]. Chinese medicine possesses significant effects on relieving breast cancer-related lymphedema, reducing cancer-related fatigue and pain, improving radiation pneumonitis and gastrointestinal side effects, protecting liver function, and even ameliorating bone marrow suppression [42]. SKO is one of the bioactive compounds from famous Chinese herbal medicine, root of Chinese herbal medicine *Arnebia euchroma* (Royle) Johnst. or *Arnebia guttata* Bunge, which has been used in traditional Chinese medicine for thousands of years. This herb is traditionally used for promoting blood circulation and removing rashes, spots as well as used for the treatment of measles impervious, sores, eczema, water and fire scald. SKO, a naphthoquinone derivative of Lithospermum erythrorhizon, is attracting more and more scientist’s research interest recent years. In aspects of treatment of breast cancers, it is reported this compound showed significant anti-tumor activity for ER + breast cancer cell lines. Mechanisms demonstrated that downregulation of ERα and GPER [44], selectively downregulation of the mRNA and enzymatic activity levels of steroid sulfatase [45], as well as specifically inhibition of pyruvate kinase-M2 in MCF-7 and many drug resistance cancer cells [46, 47], are the mainly involved molecular mechanisms for SKO induced breast cancer cell necrosis and apoptosis, which is significant different from the disclosed mechanisms of SKO induced cytotoxicity in MDA-MB-231 cell lines in our study. In triple negative breast cancer, we discovered the involvement of ubiquitination and degradation of cIAP1 and cIAP2 by SKO played a key role in cell necrosis and apoptosis (Fig. 3a, b). Taken all these findings together, it is believed that SKO has great potential for treatment of breast cancers with continuous efforts on research and development on it [48, 49].

## Conclusions

Taken together, the key finding of our study is summarized as following: SKO showed significant cytotoxicity against MDA-MB-231 cells in a concentration- and time-dependent manner, which was independent on RIP1/RIP3/MLKL signaling axis. Treatment with SKO in MDA-MB-231 cells significantly increased the autoubiquitination level of cIAP1 and cIAP2, and then facilitated the proteasome degradation of cIAP1 and cIAP2. Autoubiquitination and degradation of cIAP1 and cIAP2 induced by SKO further lead to the significant downregulation of RIP1, which played an important role in inhibition of the survival and acceleration of necrosis of MDA-MB-231 cells. Our results indicate that SKO could be a potential candidate for the treatment of triple negative breast cancer cell with continuous efforts exerted for research and development on this compound.

## Data Availability

Not applicable.

## Abbreviations

cIAP1: Cellular inhibitor of apoptosis protein 1
ELISA: Enzyme-linked immunosorbent assay
ER: Estrogen receptor
EMT: Epithelial-to-mesenchymal transition
FBS: Fetal bovine serum
HER-2: Human epidermal growth factor receptor 2
LAC: Lactacystin
MLKL: Mixed lineage kinase domain like pseudokinase
MAPK: Mitogen-activated protein kinase
MTT: 3-[4,5-Dimethylthiazol-2-yl]-2,5-diphenyl-2H-tetrazolium bromide
Nec-1: Necrostatin-1
NF-κB: Nuclear factor κB
PBS: Phosphate-buffered saline
PR: Progesterone receptor
RIP1: Receptor-interacting protein 1
RIP3: Receptor interacting protein 3
SKO: Shikonin
TNF: Tumor necrosis factor
TNFR1: TNF receptor 1
TRADD: TNF receptor 1 associated death domain
